# Comparison of Shod and Unshod Gait in Patients With Parkinson's Disease With Subthalamic and Nigral Stimulation

**DOI:** 10.3389/fnhum.2021.751242

**Published:** 2022-01-12

**Authors:** Martin A. Horn, Alessandro Gulberti, Ute Hidding, Christian Gerloff, Wolfgang Hamel, Christian K. E. Moll, Monika Pötter-Nerger

**Affiliations:** ^1^Department of Neurology, University Medical Center Hamburg-Eppendorf, Hamburg, Germany; ^2^Department of Neurophysiology and Pathophysiology, University Medical Center Hamburg-Eppendorf, Hamburg, Germany; ^3^Department of Neurosurgery, University Medical Center Hamburg-Eppendorf, Hamburg, Germany

**Keywords:** barefoot, shoes, gait, deep brain stimulation, subthalamic nucleus, substantia nigra, Parkinson's disease

## Abstract

**Background:** The Parkinsonian [i.e., Parkinson's disease (PD)] gait disorder represents a therapeutical challenge with residual symptoms despite the use of deep brain stimulation of the subthalamic nucleus (STN DBS) and medical and rehabilitative strategies. The aim of this study was to assess the effect of different DBS modes as combined stimulation of the STN and substantia nigra (STN+SN DBS) and environmental rehabilitative factors as footwear on gait kinematics.

**Methods:** This single-center, randomized, double-blind, crossover clinical trial assessed shod and unshod gait in patients with PD with medication in different DBS conditions (i.e., STIM OFF, STN DBS, and STN+SN DBS) during different gait tasks (i.e., normal gait, fast gait, and gait during dual task) and compared gait characteristics to healthy controls. Notably, 15 patients participated in the study, and 11 patients were analyzed after a dropout of four patients due to DBS-induced side effects.

**Results:** Gait was modulated by both factors, namely, footwear and DBS mode, in patients with PD. Footwear impacted gait characteristics in patients with PD similarly to controls with longer step length, lower cadence, and shorter single-support time. Interestingly, DBS exerted specific effects depending on gait tasks with increased cognitive load. STN+SN DBS was the most efficient DBS mode compared to STIM OFF and STN DBS with intense effects as step length increment during dual task.

**Conclusion:** The PD gait disorder is a multifactorial symptom, impacted by environmental factors as footwear and modulated by DBS. DBS effects on gait were specific depending on the gait task, with the most obvious effects with STN+SN DBS during gait with increased cognitive load.

## Introduction

Gait disorders with freezing of gait (FOG) remain some of the treatment-resistant symptoms in Parkinson's disease (PD) (Ebersbach et al., 2013; Armstrong and Okun, 2020), which became a focus of interest in terms of precise characterization, clinical phenomenology, treatment effects, and environmental conditions in recent years (Nutt et al., 2011). In the clinical assessment and rehabilitative setting of the Parkinsonian gait disorder, there remains one simple question regarding environmental conditions: shod or unshod gait, i.e., do they differ, and if so, which one is better in the analysis and training setting in patients with PD?

On the one hand, walking with shoes represents the most commonly used gait condition of the daily routine in patients with PD. Besides, the study used shoes as a vehicle and developed specifically designed shoes with foot-worn wearable sensors to monitor gait and posture (Martinez et al., 2018; Lee et al., 2021; Liu et al., 2021) with the option to capture gait abnormalities in everyday-life situations in PD. In addition, there were newly designed shoes with potential therapeutic use as visual cueing using laser shoes to alleviate FOG (Barthel et al., 2018a,b) or the “PDShoe” with step-synchronized vibration applied to the feet of patients with PD (Winfree et al., 2013), although some of the textured and stimulating insoles for balance and gait improvement in patients with PD seemed to have no effect (Alfuth, 2017). On the other hand, there are general discussions about the advantages of walking barefoot in younger (Cranage et al., 2020) or older people (Lord and Bashford, 1996), so that walking barefoot might be useful in the rehabilitative setting. One advantage of walking barefoot is assumed to enhance proprioceptive integration. In PD, sensorimotor deficits as tactile or proprioceptive impairments and impaired foot sole sensitivity are described (Pratorius et al., 2003; Conte et al., 2013), so that walking barefoot might be a useful rehabilitative strategy.

Beneath the rehabilitative therapeutic approaches for the PD gait disorder, there are medical and interventional therapeutic strategies as deep brain stimulation (DBS) (Nonnekes et al., 2015). DBS in the subthalamic nucleus (STN) or globus pallidus internus (GPi) improve general motor symptoms (Deuschl et al., 2006; Follett et al., 2010) and certain aspects of the hypokinetic, dopa-responsive gait disorder PD (Potter-Nerger and Volkmann, 2013); however, the long-term observations reveal residual and progressive gait symptoms (Krack et al., 2003; Potter-Nerger and Volkmann, 2013; Schlenstedt et al., 2017). As a new DBS mode to alleviate the Parkinsonian gait disorder and FOG, the combined stimulation of STN and substantia nigra (STN+SN DBS) was proposed (Weiss et al., 2011a). In a monocentric, randomized trial, STN+SN DBS was demonstrated to improve clinically FOG (Weiss et al., 2013) with a particular impact of SN-stimulation on the temporal regularization of gait integration (Scholten et al., 2017). STN+SN DBS was based on the neurophysiological consideration of dense reciprocal interconnections of substantia nigra pars reticulata (SNr) and the mesencephalic locomotor region (MLR) in the brain stem, which are involved in the control of locomotion and posture (Collomb-Clerc and Welter, 2015). It is assumed that the pathologically enhanced excitatory activity of the STN drives the SNr to excessively inhibit the MLR resulting in the decreased activation of spinal centers and consecutively impaired gait. Along this hypothesis, STN+SN DBS would functionally suppress the STN and SNr resulting in the release of the pathologically MLR inhibition and improved gait performance.

The aim of this study was 2-fold. We intended to assess, on the one hand, the effects of the rehabilitative, environmental factor “footwear” on gait and, on the other hand, the effect and possible interaction between different DBS modes, i.e., DBS of the STN (STN DBS) and STN+SN DBS, on temporal and spatial gait characteristics in patients with PD.

## Methods

### Participants

Fifteen patients (two female, age: 62.5 ± 6.7 years) suffering from moderate idiopathic PD [disease duration: 12.0 ± 5.0 years; Hoehn & Yahr stage: 2.2 ± 0.4 in the regular dopaminergic medication (MED ON) and STN DBS ON condition; Hoehn & Yahr stage: 2.6 ± 0.8 in the MED OFF condition preoperatively] participated in the study. Detailed information is shown in Table 1. No other medical or orthopedic conditions that might impact gait quality were reported in the medical history of patients with PD. Further clinical characteristics were described previously (Hidding et al., 2019).

**Table 1:** Clinical and demographic characteristics of patients with Parkinson's disease (PD).

**Case** **Gender** **Age**	**Age** **at** **onset**	**Disease duration** **(years)**	**Time with** **DBS (months)**	**LEDD (mg)**	**MoCA**	**BDI**	**PDQ 39** **OFF/STN/** **STN+SN**	**FOG** **OFF/STN/** **STN+SN**	**Berg-balance** **OFF/STN/** **STN+SN**	**UPDRS-III** **OFF/STN/** **STN+SN**	**H&Y OFF/** **STN/STN+SN**	**System**	**STN-DBS** **parameters**	**Combined** **STN+SN DBS** **parameters**	**X, Y, Z,** **coordinates**
													**Left electrode** **Right electrode**	**Left electrode** **Right electrode**	**Left electrode** **Right electrode**
1 M 61	38	23	54	1,150	27	13	36.6/28.5/29.6	2/0/0	49/54/56	32/18/30	2/2.5/2	ME	2- C+, 3.5 V, 60 μs, 125 Hz 9- 10- C+, 2.7 V, 60 μs, 125 Hz	2- C+, 3.5 V, 60 μs, 125 Hz; 0- C+, 2.0 V, 60 μs, 125 Hz 9- 10- C+, 2.7 V, 60 μs, 125 Hz; 8- C+, 2.0 V, 60 μs,125 Hz	10.9, 2.2, 4.7 10.5, 3.8, 4.7
2 M 63	40	23	105	860	26	9	46.5/25.2/25.5	2/4/0	43/52/47	41/28/29	2.5/2.5/2.5	ME	C+, 1.9 V 60 μs, 125 Hz; 2- C+, 2.9 V, 60 μs, 125 Hz 9- C+, 1.9 V, 60 μs, 125 Hz; 10- C+, 3.3 V, 60 μs, 125 Hz	2- C+, 2.9 V, 60 μs, 125 Hz; 1- 0- C+, 1.9 V (1.5 V), 60 μs, 125 Hz 10- C+, 3.3 V, 60 μs, 125 Hz; 8- 9- C+, 1.9 V (1.5 V), 60 μs, 125 Hz	11.2, 1.9, 5.6 8.3, 5.5, 4
3 M 56	47	9	36	880	26	15	21.0/26.0/28.2	14/4/1	56/54/55	39/25/30	3/2.5/2	ME	1+ 2- C+ 2.2 V, 60 μs, 125 Hz 10- C+, 4.3 V, 60 μs, 125 Hz	2-C+, 2.2 V, 60 μs, 125 Hz; 0- C+, 1.0 V, 60 μs, 125 Hz 10- C+, 4.3 V, 60 μs, 125 Hz, 8- C+, 1.0 V, 60 μs, 125 Hz	9.5, 2.8, 6.4 11.2, 1.4, 7.2
4 M 67	51	16	60	600	23	2	9.5/4.4/5.3	6/6/3	45/49/51	34/10/15	2/2/2.5	ME	C+, 1.5 V, 60 μ, 125 Hz 9- 10- C+, 3.9 V, 60 μs, 125 Hz	C+, 1.5 V, 60 μ, 125 Hz; 0- C+, 2.0 V, 60 μs, 125 Hz 9–10- C+, 3.9 V, 60 μs, 125 Hz; 8- C+, 2.0 V, 60 μs, 125 Hz	9.6, 4.7, 6.6 11.7, 3.1, 3.2
5 M 65	56	9	9	300	28	1	4.3/1.9/2.7	0/0/1	49/54/56	40/16/14	2.5/2/2	ME	C+, 2.8 V, 60 μs, 125 Hz 9- C+, 3.0 V, 60 μs, 125 Hz	C+, 2.8 V, 60 μs, 125 Hz; 0- C+, 1.5 V, 60 μs, 125 Hz 9-C+, 3.0 V, 60 μs, 125 Hz; 8- C+, 1.5 V, 60 μs, 125 Hz	10.9, 1.4, 7.7 11.1, 2.7, 6.7
6 M 74	65	9	9	360	22	1	1.6/1.0/3.8	6/0/0	55/55/56	34/23/18	2/2/2	ME	C+, 2.7 V, 130 Hz 9- C+, 2.6 V, 60 μs, 130 Hz	C+, 2.7 V, 60 μs, 125 Hz; 0- C+, 1.5 V, 60 μs, 125 Hz 9- C+, 2.9 V, 60 μs, 125 Hz; 8- C+, 1.5 V, 60 μs, 125 Hz	10.7, 2.6, 4.9 10.2, 2.5, 4.5
7 M 51	42	9	15	900	27	5	29.9/33.1/34.2	11/2/11	49/56/54	34/31/50	3/2.5/3	BS	2- 30%, 3- 70%, 3.4 mA, 60 μs, 125 Hz 10- 20%, 11- 80%, 4.0 mA, 60 μs, 125 Hz	23%, 2- 23%, 3- 54%, 4.4 mA, 60 μs, 125 Hz 9- 20%, 10- 16%, 11- 64%, 5.0 mA, 60 μs, 125 Hz	8.8, 3.4, 7.4 7.1, 4.3, 6.4
8 M 57	50	7	18	580	27	6	23.5/20.2/24.1	0/0/0	54/56/56	16/12/8	2/2/2	BS	3- 70%, 4- 30%, 4.5 mA, 60 μs, 130 Hz 12- 100%, 3.8 mA, 60 μs, 130 Hz	3- 61%, 4- 26%, 1- 13%, 5.2 mA, 60 μs, 130 Hz 12- 85%, 9- 15%, 4.5 mA, 60 μs, 130 Hz	11.9, 3.4, 6.1 11.6, 2.7, 5.9
9 M 71	61	11	13	600	27	11	37.0/50.6/41.8	4/3/1	53/53/54	34/27/16	2.5/2/2	ME	C+, 3.5 V, 60 μs, 125 Hz 9- C+, 2.7 V, 60 μs, 125 Hz	C+, 3.5 V, 60 μs, 125 Hz; 0- C+, 1.0 V, 60 μs, 125 Hz 9- C+, 2.7 V, 60 μs, 125 Hz; 8- C+, 1.0 V, 60 μs, 125 Hz	1.3, 2.2, 6.2 12.2, 0.2, 5.2
10 M 66	54	13	6	300	22	10	25.2/38.9/33.2	0/0/0	56/54/54	51/16/14	2.5/2.5/2	ME	C+, 3.8 V, 60 μsec, 130 Hz 9- C+, 3.6 V, 60 μz 130 Hz	3.8 V 60 μsec 125 Hz 0- 1.0 V 60 μsec, 125 Hz 9- C+ 3.6 V, 60 μsec, 125 Hz 8- C+ 1,0 V 60 μsec, 125 Hz	11.2, 6.5, 6.6 10.5, 4.2, 5.1
11 F 66	56	10	5	440	25	2	6.8/5.0/4.2	4/0/0	54/56/55	27/7/10	2/2.5/2.5	BS	5-6-7- (Ring) C+, 2.2 mA, 60 μsec, 130 Hz 13-14-15- (Ring) C+, 2.4 mA 60 μsec, 130 Hz	5- (23%) 6- (23%) 7- (23%) 1- (31%) C+, 2.9 mA, 60 μsec, 130 Hz 13-(24%) 14- (23%) 15-(23%) 9-(30%) C+, 3.1 mA, 60 μsec, 130 Hz	10.9, 2.5, 5.7 10.4, 0.44, 5.2
12 F 66	57	9	5	700	25	13	30.4	18	55	42	2	ME	Withdrawal in phase I, experimental phase II not performed	2- C+, 2.4 V, 60 μs, 125 Hz; 0- C+, 0.7 V, 60 μs, 125 Hz 11- C+, 2.5 V, 60 μs, 125 Hz; 8- C+, 0.7 V, 60 μs, 125 Hz	10.2, 0.9, 5.2 9.0, 0.6, 7.8
13 M 55	42	13	23	700	28	5	13.3	5	56	28	2	ME	Withdrawal in phase I, experimental phase II not performed	3- C+, 2.9 V, 60 μs, 125 Hz; 0- C+, 0.7 V, 60 μs, 125 Hz 10- C+, 2.9 V, 60 μs, 125 Hz 8- C+, 0.7 V, 60 μs, 125 Hz	9.2, 2.8, 7.7 10.2, 2.4, 6
14 M 53	43	10	16	860	24	8	14.7	0	55	20	2.5	BS	Withdrawal in phase I, experimental phase II not performed	3-C+ 2,7 mA, 60 μs, 119 Hz; 1- C+, 0.7 mA, 60 μs, 119 Hz 12-/13- C+, 4,7 mA, 60 μs, 119 Hz; 9- C+, 0.7 mA, 60 μs, 119 Hz	10.7, 5.3, 6.9 7.7, 3.1, 6.8
15 M 66	57	9	5	590	28	5	6.8	9	56	34	2	BS	Withdrawal in phase I, experimental phase II not performed	13- (16%) 14- (45%) 15- (16%) 9- (23%) 4.5 mA, 60 μsec, 130 Hz 5- (29%) 6-(15%), 7- (29%), 1- (27%), 3.7 mA, 60 μsec, 130 Hz	11.6,4.1, 7.4 10.9, 2.2,6.2

*“Disease duration (years)” is calculated from the date of the first diagnosis to the date of baseline measurement of the experiment. Electrode coordinates are given in relation to the anterior and posterior commissure (AC-PC) line (mm) lateral to the midline (X), posterior to the midcommissural point (Y), and inferior to the intercommissural plane (Z). Notably, the deepest contacts were contact 0 and 8 (Medtronic) or contact 1 and 9 (Boston Scientific). LEDD, levodopa equivalent daily dose; ME, Medtronic; BS, Boston Scientific; MoCA, Montreal Cognitive Assessment score; BDI-I, Becks Depression Inventory; Berg Balance scale sum score; short form of the Berg Balance scale comprehending only items 1, 6, 8, 9, 10, 13, 14; FOG, Freezing of Gait Assessment Course score; UPDRS-III, motor-subscore (part III) of Unified Parkinson's Disease Rating Scale of the Movement Disorder Society; H&Y, Hoehn & Yahr scale; NA, not applicable*.

Patients with PD were included if (1) bilateral electrode implantation in the STN for DBS was performed at least 5 months before, (2) the deepest contacts of the implanted electrodes were positioned within the dorsal aspect of the SN along image-based electrode reconstruction (location of the electrode tip at least 4.5–6 mm inferior to AC-PC line), and (3) dopaminergic medication and stimulation parameters were unchanged in the preceding 4 weeks before baseline measurements. Notably, 10 patients with PD were implanted with Medtronic DBS systems (model 3389; Medtronic, Minneapolis, MN, USA), and five patients with 8-poled electrodes from Boston Scientific (Valencia, CA, USA). Preoperatively, all patients with PD were screened and selected for DBS surgery in accordance with the common guidelines of DBS surgery [Core Assessment Program for Surgical Interventional Therapies (CAPSIT) protocol (Defer et al., 1999)]. Patients showed significant improvement in the motor subscore (part III) of the Movement Disorder Society (MDS)-Unified Parkinson's Disease Rating Scale (UPDRS) after the intake of immediate-release soluble levodopa (MED OFF: 38.0 ± 17.7, MED ON: 12.0 ± 8.4, improvement of 67%). The daily levodopa-equivalent dose decreased from 990.3 ± 205.8 mg preoperatively to 654.7 ± 245.7 mg postoperatively. Four patients withdrew from the study during STN+SN DBS mode due to side effects such as general uncomfortable feeling, increased confusion, hallucinations, aggressiveness, and a lack of beneficial effects of levodopa intake. We also evaluated 11 healthy individuals who were matched by gender (two females), age (64 ± 6.8 years for controls vs. 62.5 ± 6.7 years for PD patients), and the Montreal Cognitive Assessment (MoCA) (Gill et al., 2008) score (28.5 for controls vs. 25.5 for PD patients).

### Design

**Figure 1:**
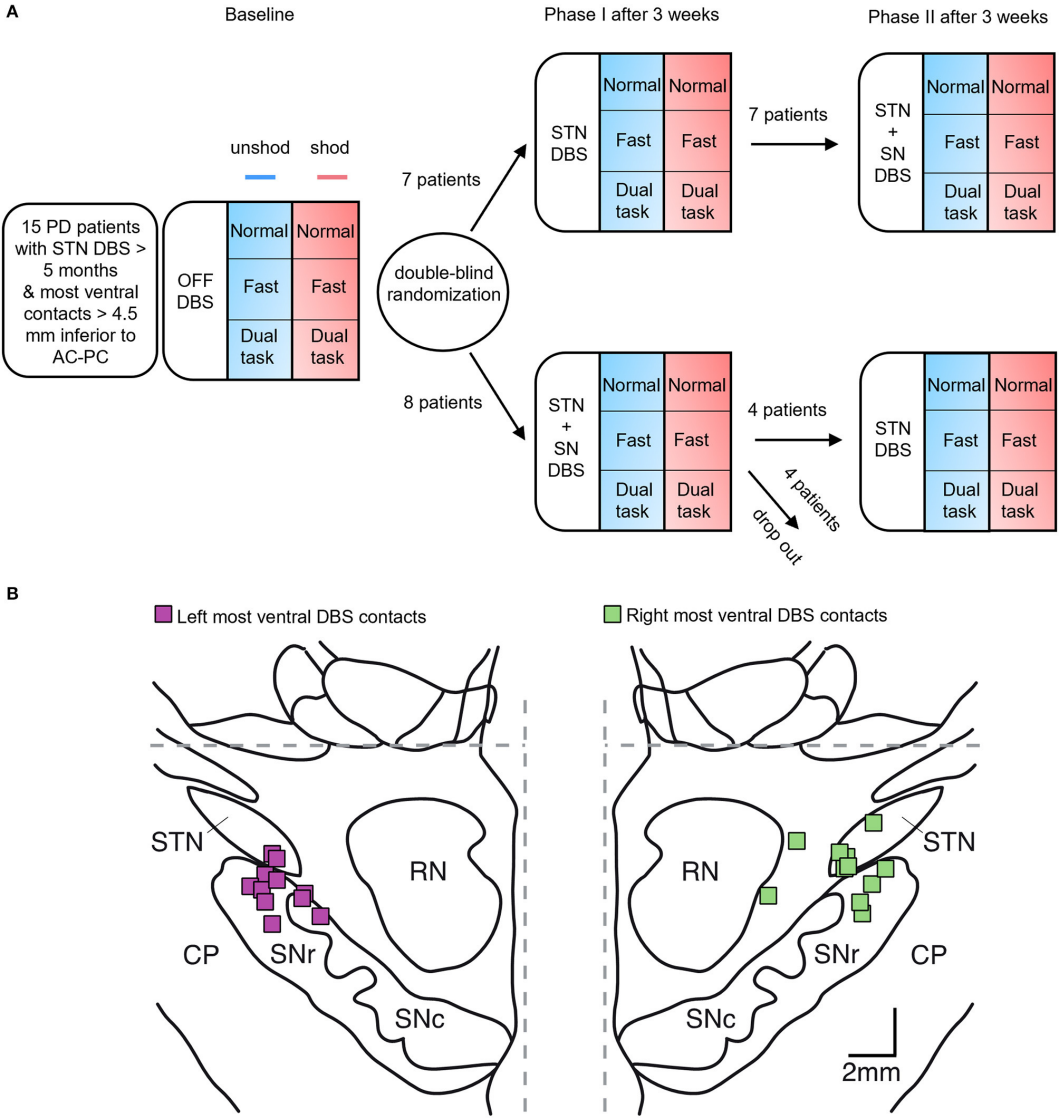
Experimental setting. **(A)** Study design: randomized crossover trial over 6 weeks. At baseline, patients were evaluated in the STIM OFF mode. Spatiotemporal parameters of gait were measured under six paradigms in randomized order: the three gait tasks were performed at normal pace, at a fast pace, and in a dual-task setting either shod (red squares) or unshod (blue squares), followed by the other footwear condition. After baseline assessment, patients with Parkinson's disease (PD) were assigned in phase I to either conventional deep brain stimulation of the subthalamic nucleus (STN DBS) or combined STN and substantia nigra (STN+SN) stimulation in a double-blind randomized order. After 3 weeks, patients were switched to the other stimulation mode for the next 3 weeks. Of note, four patients with PD programmed in STN+SN DBS withdrew within the first week and terminated the study prematurely. **(B)** stereotactic reconstruction of 11 patients included in statistical analysis: squares indicate the localization of the most ventral DBS electrode contacts for the left and right hemisphere, on frontal sections of the stereotactic atlas of Morel (2007), at a level 5 mm behind midcommissural point. Interrupted gray lines denote midline and anterior and posterior commissure (AC-PC) level, respectively. CP, cerebral peduncle; RN, red nucleus; SNr, substantia nigra pars reticulata; SNc, substantia nigra pars compacta; STN, subthalamic nucleus.

The project was a single-center, randomized, double-blind, crossover clinical trial at the departments of neurology and neurosurgery at the University Medical Center Hamburg-Eppendorf (UKE) to compare the effect of STN stimulation vs. STN+SN DBS in patients with PD as described previously (Hidding et al., 2017) (Figure 1). In this study, we compared temporal and spatial characteristics of gait while walking barefoot or with shoes during STN+SN DBS, conventional STN DBS, or no stimulation (STIM OFF) in patients with PD.

At baseline, we did a monopolar review of the most ventral contacts located in the SN. Thresholds with side effects were 3.3 ± 0.9 mA (range: 2.0–5.0 mA) in left SN and 3.3 ± 1.1 mA (range: 1.5–5.0 mA) in right SN. The stimulation strength of at least 0.5 mA below the individual side effect threshold was chosen, which was in the range given in the literature (Weiss et al., 2013). The average stimulation parameters in SN were 1.2 ± 0.5 mA (range: 0.7–2 mA) applied symmetrically on either side. At phase I, patients with PD were evaluated and then randomized to conventional STN DBS or STN+SN DBS. Phase II started 3 weeks after, with crossover reprogramming for the following 3 weeks. There was no washout period in between the two phases (Figure 1). All visits were performed with MED ON, which was kept constant throughout the whole course of the study. Stimulation parameters were fixed during phase I and phase II of the study, besides in one patient, in which stimulation amplitude in the SN had to be reduced after 2 days due to dyskinesias (Hidding et al., 2019).

The study visits took place at the university hospital regularly in the morning. The patients had taken the last levodopa dosage at home.

To assess gait kinematics of controls and patients with PD, we used the GAITRite^®^ Walkway System. The duration of all gait task performances for gait analysis was 27.4 ± 5.3 min. The GAITRite^®^ consists of a walkway with the overall dimensions of 90 cm × 7 m × 3.2 mm. We analyzed the *temporal* parameters as velocity (cm/s), cadence (steps per minute), single support (percentage of the gait cycle time of the same foot), and the coefficient of variation (CV) of the stride time (Hausdorff et al., 1998) as well as the *spatial* parameters as step length (cm) and base width (cm) (Bilney et al., 2003). To evaluate gait asymmetry, we calculated the step length symmetry ratio (i.e., the ratio of the mean step length of best and worst side).

During each assessment, participants were asked to walk over the GAITRite^®^ Walkway System performing three different gait tasks as follows: (1) straightforward gait at self-paced, normal walking speed, (2) straightforward gait with fast walking speed, and (3) gait with the increased cognitive load as dual-task performance (DT) when patients walked while performing a mental arithmetic task, turning at the end of the walkway and walking back. Each gait task was performed while wearing shoes and barefoot; for each task, the walk was repeated three times. For a better comparison between different gait tasks, we calculated gait metrics in the DT scenario using only the first straightforward part of the task.

### Implantation of the Permanent DBS Electrodes

The DBS electrode placement was guided by intraoperative microelectrode recording (MER) and test stimulation. Three parallel tracks were used to map the subthalamic region with tungsten electrodes (NeuroProbe electrodes, Alpha Omega Inc., Nazareth, Israel; impedance: 685 ± 245 kOhm). The subthalamic sensorimotor region was identified by cell responses to passive and active movements and a high prevalence of oscillating neuronal activities in the beta-frequency range (13–30 Hz). The differentiation of STN from SN was based on the established electrophysiological criteria (Sharott et al., 2014; Hidding et al., 2017). The optimal target site for electrode implantation was further determined by the clinical evaluation of macrostimulation responses (Moll et al., 2014; Potter-Nerger et al., 2017).

### Stereotactic Reconstruction of Most Ventral Electrode Contacts

The reconstruction of the active DBS lead contacts (electrode model 3389, Medtronic, Minneapolis, MN, USA, in 8 cases, and electrode model 2201 and model 2202, Boston Scientific, Valencia, CA, USA, in 2 cases and 1 case, respectively) was performed by the co-registration of the preoperative T1 MRI scans and postoperative CT scans using iPlan (iPlan Stereotaxy; Brainlab, Feldkirchen, Germany). Further details concerning the localization of active electrode contacts were reported previously (Hamel et al., 2003; Fischer et al., 2016; Hidding et al., 2017). According to stereotactic atlases, high-resolution MRI, and MER-guided mapping, the upper border of the SNr is positioned 4.5–6 mm below the plane in between anterior and posterior commissure (AC and PC; Figure 1; Table 1) (Weiss et al., 2013).

### Statistics

Since four patients withdrew from the study due to intolerance of STN+SN DBS, analyses were performed in the remaining 11 patients completing the whole course of the study.

In a first step, we compared age-matched, healthy controls and patients with PD in the STIM OFF condition by analyzing two-way repeated-measures ANOVAs with the intrasubject factors such as 1. footwear (barefoot or shoes) and 2. gait task (normal gait, fast gait, and dual task) and with the intersubject factor group (controls vs. patients with PD in STIM OFF).

In a second step, we assessed the effect of DBS by performing three-way repeated-measures ANOVAs with the intrasubject factors: 1. footwear (barefoot or shoes), 2. stimulation condition (STIM OFF, STN DBS, and combined stimulation STN+SN DBS), and 3. gait task (normal gait, fast gait, and dual task) for gait kinematics.

Greenhouse–Geisser-corrected *p*-values were calculated if the violation of sphericity was obvious in Mauchly's sphericity test. Alpha level was set at 0.05. *Post hoc* Wilcoxon signed-ranks tests were performed to compare the effects of different stimulations or gait tasks (IBM SPSS Statistics version 25.0, SPSS, Inc., Chicago, IL, USA).

In a third step, *post hoc* repeated-measures correlations were performed using the rmcorr R package (R version 3.5.0; rmcorr package) (Bakdash and Marusich, 2017). This method was applied to assess consistencies between the gait parameters and the clinical scores at the three DBS stimulation conditions.

## Results

### The Effect of Footwear on Gait Kinematics in Controls and Patients With PD

Shod or unshod gait induced distinct changes of gait characteristics in healthy controls and patients with PD in STIM OFF (Table 2). Of note, baseline gait characteristics between the two groups differed. As expected, in healthy controls, gait velocity was higher (*p* = 0.003), step length (*p* = 0.001) and relative single support time (*p* = 0.033) were longer compared to patients with PD, whereas gait asymmetry (*p* = 0.052) and gait variability (*p* = 0.006) were smaller compared to patients with PD in different gait tasks. During fast gait, cadence (*p* = 0.004) was higher in healthy controls compared to PD, while base width (*p* = 0.029) was smaller in the dual-task scenario in controls compared to patients with PD.

**Table 2:** Results of three-way repeated-measures ANOVAs.

			**Unshod**				**Shod**				**Within-subjects contrasts for controls**			**Within-subjects contrasts for patients with PD**				
**Gait** **parameter**		**Gait task**	**Controls**	**OFF**	**STN**	**STN+SN**	**Controls**	**OFF**	**STN**	**STN+SN**	**Unshod vs. shod**	**Dual vs. normal**	**Normal vs. fast**	**Unshod vs. shod**	**OFF vs. STN**	**STN vs. STN+SN**	**Dual vs. Normal**	**Normal vs. Fast**
Velocity	← Pace	Dual	111.2 ± 16.7	82.8 ± 30.6	90.8 ± 25.5	95.3 ± 27.8	119.4 ± 21.4	83.5 ± 25.7	96.4 ± 28.7	101.4 ± 27.7	***F*** **= 16.31**	***F*** **= 23.39**	***F*** **= 88.33**	*F* = 0.029	*F* = 0.72	*F* = 2.91	***F*** **= 20.55**	***F*** **= 159.99**
		Normal	131.8 ± 13.4	108.5 ± 17.7	110.1 ± 23.3	118.4 ± 21.1	142.0 ± 15.2	112.1 ± 18.8	114.5 ± 18.9	119.5 ± 22.3	***p*** **= 0.002**	***p*** **= 0.001**	***p*** **< 0.001**	*p* = 0.869	*p* = 0.417	*p* = 0.119	***p*** **= 0.001**	***p*** **< 0.001**
		Fast	189.4 ± 25.5	155.8 ± 24.8	156.1 ± 26.3	165.6 ± 29.1	203.6 ± 27.8	150.1 ± 26.6	151.7 ± 20.4	160.0 ± 20.0	**η^2^ = 620**	**η^2^ = 0.701**	**η^2^ = 0.898**	η^2^ = 0.003	η^2^ = 0.067	η^2^ = 0.225	**η^2^ = 0.673**	**η^2^ = 0.941**

Step length	← Pace	Dual	62.7 ± 5.1	49.9 ± 11.1	55.6 ± 10.5	55.6 ± 10.5	67.4 ± 6.5	53.1 ± 9.0	58.0 ± 8.0	61.7 ± 8.9	***F*** **= 70.59**	***F*** **= 35.54**	***F*** **= 57.13**	***F*** **= 15.88**	*F* = 0.65	***F*** **= 8.14**	***F*** **= 26.68**	***F*** **= 61.61**
		Normal	68.9 ± 5.4	58.7 ± 5.8	59.5 ± 7.6	62.9 ± 7.5	76.4 ± 5.5	63.6 ± 5.4	63.8 ± 5.0	67.0 ± 7.2	***p*** **< 0.001**	***p*** **< 0.001**	***p*** **< 0.001**	***p*** **= 0.003**	*p* = 0.440	***p*** **= 0.017**	***p*** **< 0.001**	***p*** **< 0.001**
		Fast	80.4 ± 7.2	70.4 ± 6.9	69.8 ± 5.5	72.6 ± 6.3	86.1 ± 7.6	74.2 ± 6.2	72.8 ± 4.4	77.6 ± 5.1	**η^2^ = 0.876**	**η^2^ = 0.780**	**η^2^ = 0.851**	**η^2^ = 0.614**	η^2^ = 0.061	**η^2^ = 0.449**	**η^2^ = 0.727**	**η^2^ = 0.727**

Cadence	← Pace	Dual	106.19 ± 9.6	97.4 ± 20.0	101.6 ± 17.5	101.8 ± 16.0	105.4 ± 13.4	93.3 ± 20.4	98.3 ± 19.3	97.8 ± 18.4	*F* = 0.66	***F*** **= 6.05**	***F*** **= 76.25**	***F*** **= 13.64**	*F* = 0.72	*F* = 0.63	***F*** **= 9.78**	***F*** **= 93.16**
		Normal	114.1 ± 7.7	110.9 ± 13.4	110.4 ± 14.6	112.7 ± 11.9	111.9 ± 6.1	105.5 ± 12.9	107.3 ± 12.5	106.7 ± 12.6	*p* = 0.436	***p*** **= 0.034**	***p*** **< 0.001**	***p*** **= 0.004**	*p* = 0.417	*p* = 0.808	***p*** **= 0.011**	***p*** **< 0.001**
		Fast	142.6 ± 14.5	132.5 ± 13.9	133.6 ± 14.7	136.7 ± 16.3	141.9 ± 13.7	120.7 ± 14.2	124.6 ± 11.8	123.8 ± 13.8	η^2^ = 0.062	**η^2^ = 0.377**	**η^2^ = 0.884**	**η^2^ = 0.577**	η^2^ = 0.067	η^2^ = 0.006	**η^2^ = 0.494**	**η^2^ = 0.903**

Single support	← Pace	Dual	37.3 ± 1.7	35.3 ± 2.9	36.4 ± 2.3	37.2 ± 2.0	36.3 ± 2.1	33.6 ± 1.9	35.1 ± 1.9	35.8 ± 1.6	***F*** **= 13.99**	***F*** **= 38.73**	***F*** **= 179.96**	***F*** **= 35.73**	*F* = 3.19	***F*** **= 8.43**	***F*** **= 35.31**	***F*** **= 134.9**
		Normal	38.8 ± 1.0	38.0 ± 1.5	38.4 ± 1.7	39.1 ± 1.4	37.9 ± 1.9	36.5 ± 1.4	36.7 ± 1.5	36.8 ± 1.6	***p*** **= 0.004**	***p*** **< 0.001**	***p*** **< 0.001**	***p*** **< 0.001**	*p* = 0.105	***p*** **= 0.016**	***p*** **< 0.001**	**3p < 0.001**
		Fast	41.6 ± 1.1	40.6 ± 1.6	40.7 ± 1.7	41.4 ± 1.5	40.3 ± 1.8	38.5 ± 1.8	38.3 ± 1.9	38.9 ± 1.7	**η^2^ = 0.583**	**η^2^ = 0.795**	**η^2^ = 0.947**	**η^2^ = 0.781**	η^2^ = 0.242	**η^2^ = 0.457**	**η^2^ = 0.779**	**η^2^ = 0.931**

CV of std of stride time	← Pace	Dual	3.3 ± 1.1	6.6 ± 2.5	5.7 ± 1.8	5.8 ± 2.0	2.8 ± 1.4	7.2 ± 4.5	5.2 ± 1.6	5.6 ± 2.4	***F*** **= 11.45**	***F*** **= 7.28**	***F*** **= 5.68**	*F* = 2.98	*F* = 2.25	*F* = 0.92	***F*** **= 12.33**	*F* = 0.72
		Normal	2.4 ± 0.5	4.2 ± 1.6	4.3 ± 1.6	4.6 ± 1.7	1.9 ± 0.7	3.9 ± 1.0	3.5 ± 1.3	3.6 ± 1.0	***p*** **= 0.007**	***p*** **= 0.022**	***p*** **= 0.038**	*p* = 0.115	*p* = 0.164	*p* = 0.361	***p*** **= 0.006**	*p* = 0.415
		Fast	3.3 ± 1.2	4.5 ± 1.8	4.4 ± 1.5	4.9 ± 2.0	2.7 ± 1.1	3.5 ± 1.3	4.1 ± 1.1	3.8 ± 1.2	**η^2^ = 0.534**	**η^2^ = 0.421**	**η^2^ = 0.362**	η^2^ = 0.230	η^2^ = 0.184	η^2^ = 0.084	**η^2^ = 0.552**	η^2^ = 0.067

Step length symmetry ratio	← Pace	Dual	1.03 ± 0.03	1.09 ± 0.07	1.05 ± 0.04	1.04 ± 0.04	1.03 ± 0.04	1.12 ± 0.08	1.04 ± 0.02	1.04 ± 0.04	*F* = 0.02	*F* = 0.01	*F* = 4.65	*F* = 0.01	***F*** **= 10.72**	*F* = 0.017	***F*** **= 5.93**	*F* = 0.03
		Normal	1.03 ± 0.02	1.06 ± 0.05	1.04 ± 0.03	1.04 ± 0.04	1.03 ± 0.02	1.05 ± 0.03	1.04 ± 0.03	1.05 ± 0.04	*p* = 0.883	*p* = 0.951	*p* = 0.056	*p* = 0.942	***p*** **= 0.008**	*p* = 0.899	***p*** **= 0.035**	*p* = 0.855
		Fast	1.04 ± 0.03	1.06 ± 0.028	1.06 ± 0.03	1.05 ± 0.02	1.03 ± 0.02	1.06 ± 0.04	1.04 ± 0.02	1.04 ± 0.03	η^2^ = 0.002	η^2^ = 0.000	η^2^ = 0.318	η^2^ = 0.001	**η^2^ = 0.517**	η^2^ = 0.002	**η^2^ = 0.372**	η^2^ = 0.004

Base width	← Pace	Dual	10.09 ± 2.2	13.8 ± 5.8	12.8 ± 5.6	13.8 ± 5.7	9.7 ± 2.3	14.2 ± 6.1	12.2 ± 4.3	13.1 ± 5.2	*F* = 0.03	*F* = 0.27	*F* = 0.53	*F* = 1.26	*F* = 3.09	*F* = 1.22	***F*** **= 10.58**	*F* = 0.28
		Normal	9.8 ± 1.6	11.7 ± 3.9	11.3 ± 3.3	11.1 ± 3.4	9.5 ± 1.2	12.0 ± 4.0	10.8 ± 3.7	11.3 ± 3.8	*p* = 0.864	*p* = 0.614	*p* = 0.485	*p* = 0.288	*p* = 0.109	*p* = 0.295	***p*** **= 0.009**	*p* = 0.610
		Fast	9.5 ± 1.9	11.4 ± 5.0	11.0 ± 4.1	11.4 ± 4.2	10.1 ± 1.4	11.3 ± 4.1	10.4 ± 3.7	11.4 ± 3.4	η^2^ = 0.003	η^2^ = 0.026	η^2^ = 0.050	η^2^ = 0.112	η^2^ = 0.236	η^2^ = 0.109	**η^2^ = 0.514**	η^2^ = 0.027

*Comparison of gait parameters walking barefoot and with shoes during the three gait tasks under the three stimulation conditions. The stimulation conditions were as follows: OFF, DBS switched off; STN, conventional deep brain stimulation of the subthalamic nucleus (STN DBS); STN+SN, combined STN+SN DBS. Gait tasks were as follows: Dual, gait during the dual task; Normal, normal gait; Fast, fast gait. Values reported are mean ± SD calculated for both legs. The p-values < 0.05 are highlighted in bold*.

To evaluate the effect of footwear in different gait tasks in both groups in detail, two-way repeated-measures ANOVAs with the intrasubject factors such as 1. footwear and 2. gait task and with the intersubject factor group (control vs. PD in STIM OFF) were performed.

The factor footwear impacted *gait velocity* only in healthy controls (footwear × subject interaction: *F* = 4.56, *p* = 0.045, η^2^ = 0.186) with increased gait speed with shoes during normal and fast gait tasks. *Gait velocity* was modulated by gait task (*F* = 138.15, *p* < 0.001, η^2^ = 0.874) in all subjects, with the highest speed in the fast gait task (*p* < 0.001) and slowest gait speed in the DT (*p* = 0.003) compared to normal gait.

*Step length* was significantly impacted by footwear (*F* = 40.54, *p* < 0.001, η^2^ = 0.670) in all subjects with larger step lengths with shoes (*p* = 0.003) and smaller step lengths when walking barefoot. Gait task impacted step length (*F* = 99.19, *p* < 0.001, η^2^ = 0.832) with larger steps during fast gait (*p* < 0.001) and smaller steps during DT (*p* = 0.001) compared to normal gait. As already shown in previous studies, step length was higher in healthy controls compared to patients with PD in different gait tasks.

*Cadence* was significantly affected by footwear (*F* = 9.24, *p* = 0.006, η^2^ = 0.316) through all gait conditions, which was particularly obvious in patients with PD (footwear × subject interaction: *F* = 4.58, *p* = 0.045, η^2^ = 0.186) with a significantly lower cadence during shod gait and higher cadence when walking barefoot (*p* = 0.018). The gait task also affected cadence (*F* = 86.75, *p* < 0.001, η^2^ = 0.813) with higher cadence during fast gait and lower cadence during DT compared to normal gait in all subjects.

The relative *single support* (as a percentage of gait cycle time) was significantly modulated by footwear (*F* = 24.59, *p* < 0.001, η^2^ = 0.551). In controls, the relative single support time was longer than in patients with PD. As expected, the gait task (*F* = 121.16, *p* < 0.001, η^2^ = 0.858) influenced the relative single support time with prolongation during fast gait (*p* < 0.001) and reduction while DT (*p* = 0.032) compared to a normal walk in all subjects.

The temporal *gait variability* as measured by the CV of the stride time was not affected by footwear in any gait task. However, gait variability changed within different gait tasks (*F* = 10.34, *p* < 0.001, η^2^ = 0.341) particularly in patients with PD (gait task × subject interaction: *F* = 5.44, *p* = 0.021, η^2^ = 0.214) with the highest gait variability during DT (*p* = 0.017) compared to normal or fast gait (*p* = 0.061).

The *asymmetry index* of the step length was not affected by the factor footwear in different gait tasks in any subjects. As expected, gait asymmetry was in principal lower in controls compared to patients with PD (*p* = 0.045). There was a group-dependent effect of the factor gait task (gait task × subject interaction: *F* = 4.37, *p* = 0.019, η^2^ = 0.179) with a significant increase of gait asymmetry during DT compared to normal gait in patients with PD, which was not obvious in controls.

The gait *base width* was principally smaller in controls compared to patients with PD (*p* = 0.042). Base width was not modulated by footwear in any subject but modulated by gait task (*F* = 7.54, *p* = 0.004, η^2^ = 0.274), particularly in patients with PD with a broad-based gait during DT (gait task × subject interaction: *F* = 6.0, *p* = 0.01, η^2^ = 0.231).

### The Effect of DBS on Shod and Unshod Gait Kinematics in Patients With PD

**Figure 2:**
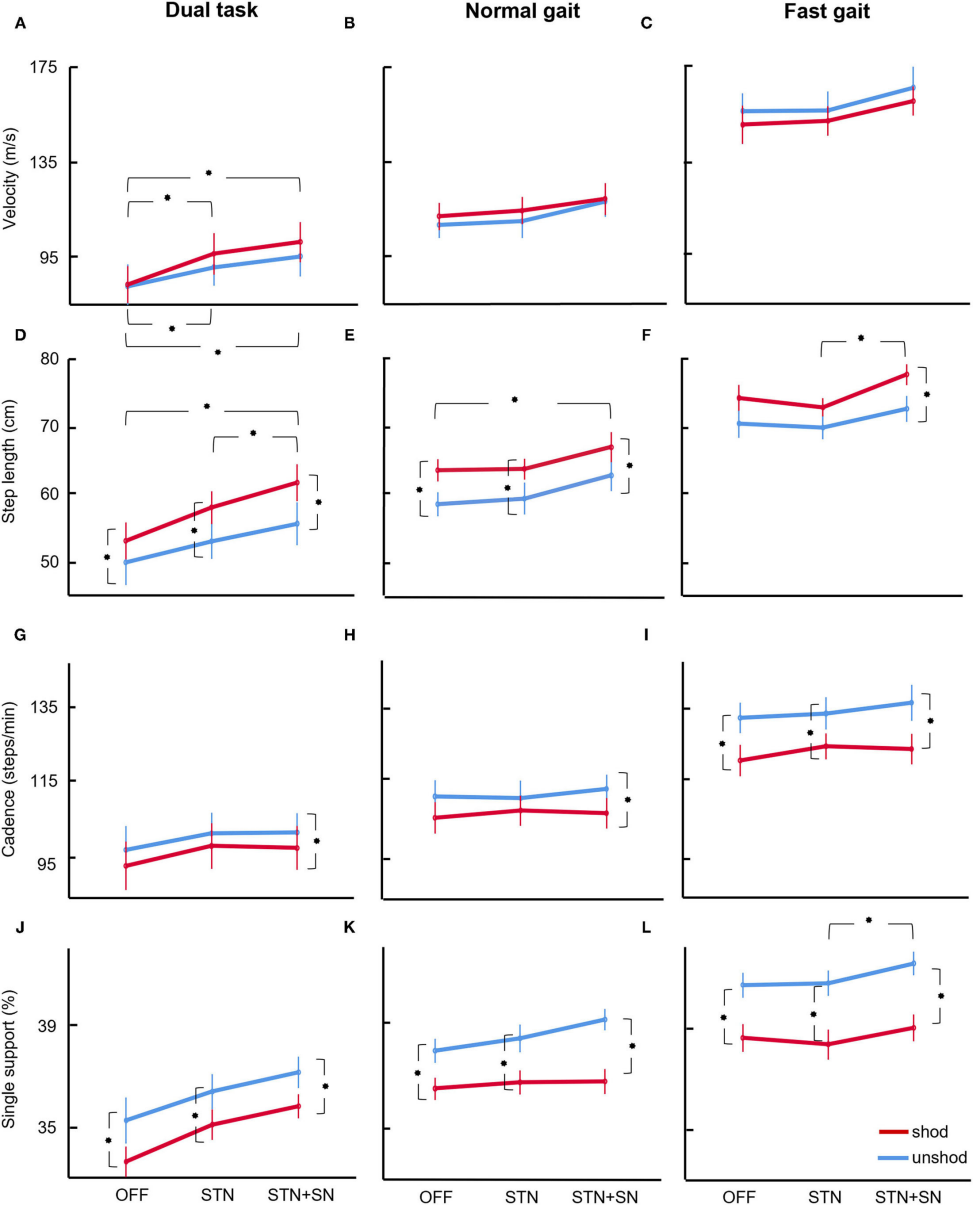
ANOVA results. Results for ANOVA on gait parameters velocity (panels **A–C**), step length (panels **D–F**), cadence (panels **G–I**), and single support (panels **J–L**) are shown for patients with PD OFF DBS, and under STN or STN+SN DBS for the paradigms normal gait, fast gait, and dual task. Line plots are ranked by task pace, i.e., from the slowest pace during the dual task **(left)** to the fastest pace during the fast gait task **(right)**. Values reported are mean and SE bars. Stars denote significant *post hoc* Wilcoxon signed-ranks tests between gait parameters in the unshod and shod conditions and in the three stimulation conditions (*p* < 0.05).

The DBS affected certain gait kinematics in patients with PD in different gait tasks. Three-way repeated-measures ANOVAs with the intrasubject factors such as 1. footwear, 2. stimulation, and 3. gait task revealed the main finding of the DBS-specific effects on gait kinematics particularly in gait tasks with increased cognitive load were recorded. Findings are shown in Figure 2 and Table 2.

*Gait velocity* was significantly modulated during different gait tasks (*F* = 103.91, *p* < 0.001, η^2^ = 0.912) with increased gait speed during the fast gait task and slower speed during DT compared to normal gait. DBS seemed to change gait velocity (*F* = 2.86, *p* = 0.081, η^2^ = 0.223), but the effect was not significant. Gait velocity was not affected by footwear throughout all tasks.

*Step length* was significantly impacted by DBS (*F* = 5.62, *p* = 0.012, η^2^ = 0.360) with significant interaction with gait task (*F* = 3.69, *p* = 0.012, η^2^ = 0.270), indicating gait task-dependent step length increment. We observed a significantly higher step length during STN+SN DBS compared to STIM OFF (*p* = 0.019) and STN DBS (*p* = 0.032), particularly during DT. Thus, STN+SN DBS improved specifically step length in the gait task with increased cognitive load. As in untreated patients with PD in STIM OFF, we observed, in DBS conditions, an effect of footwear (*F* = 15.88, *p* = 0.003, η^2^ = 0.614) with larger step lengths with shoes and smaller step lengths when walking barefoot. There were no significant interactions of footwear with DBS condition or gait task, indicating an overall similar effect of footwear-related step length increment across all stimulation conditions and gait tasks. Gait task impacted step length (*F* = 53.35, *p* < 0.001, η^2^ = 0.842) with larger steps during fast gait and smaller steps during DT compared to normal gait.

*Cadence* was not significantly modulated by the DBS stimulation mode, but there was a significant effect of gait task (*F* = 57.375, *p* < 0.001, η^2^ = 0.852) and footwear (*F* = 13.64, *p* = 0.004, η^2^ = 0.577) with a significantly lower cadence during shod gait and higher cadence when walking barefoot. This decrease in cadence by wearing footwear was an overall effect across all stimulation or gait tasks since interactions within the model were not significant.

The relative *single support* (as percentage of gait cycle time) was modulated by all three factors, by DBS (*F* = 7.62, *p* = 0.003, η^2^ = 0.432), footwear (*F* = 35.75, *p* < 0.001, η^2^ = 0.781), and gait task (*F* = 81.43, *p* < 0.001, η^2^ = 0.891). As expected, the single support was modulated through the gait tasks with prolongation during fast gait and reduction while DT compared to a normal walk. In contrast, DBS lengthened single support, particularly during STN+SN DBS compared to STIM OFF (*p* = 0.027) and STN DBS (*p* = 0.047). This single support extension was depending on the gait task (DBS × gait task interaction: *F* = 5.26, *p* = 0.017, η^2^ = 0.345) with the most obvious findings during DT.

The temporal *gait variability* as measured by the CV of the stride time was not significantly affected by DBS or footwear. However, gait variability changed within different gait tasks (*F* = 8.96, *p* = 0.008, η^2^ = 0.473) with the highest gait variability during DT compared to normal or fast gait.

The *asymmetry index* of the step length was not affected by the factor footwear or gait task. However, DBS impacted gait symmetry significantly (*F* = 5.02, *p* = 0.017, η^2^ = 0.334), particularly in specific gait tasks (DBS × gait task interaction: *F* = 4.98, *p* = 0.029, η^2^ = 0.332). DBS within STN and STN+SN improved and reduced gait asymmetry compared to STIM OFF, particularly in the DT (*p* = 0.057).

The gait *base width* was only significantly modulated by gait task (*F* = 9.78, *p* = 0.001, η^2^ = 0.495) but not by DBS or footwear. In the DT, the base width was widened, indicating a more unstable gait pattern compared to a normal or fast gait.

Gait characteristics of objective gait analyses as single support time, step length, and velocity correlated with the clinical scores of FOG, balance, and motor scores, particularly in the DT condition underlining the close relationship of objective gait metrics and clinical scores (Supplementary Figure 1).

## Discussion

In this study, we found the modulation of gait kinematics by footwear and DBS within the specific gait tasks in patients with PD. Footwear impacted gait characteristics in patients with PD with longer step length and lower cadence throughout all DBS conditions and gait tasks. In contrast, STN DBS and STN+SN DBS induced circumscribed changes of certain gait parameters depending on the specific gait task. DBS induced step length increment, gain of relative single support time, and reduction of gait asymmetry depending on the gait task. These changes were particularly obvious during STN+SN DBS in DT conditions, thus in gait tasks with increased cognitive load.

There are limitations to the study. The sample size of patients with PD was small since, during surgery processes for conventional STN DBS, the most caudal electrode contact reaches the SN only in a few patients. We decided to evaluate the patients in daily MED ON conditions to assess patients with PD in the everyday condition; however, we might have ceiling effects and miss further differences between different DBS conditions. Another limitation might be a lack of the use of a standardized shoe in all patients; the patients were asked to wear their own, comfortable outdoor shoes. Besides, gait analyses on the GAITRite^®^ carpet offered short time stamps of the gait performance in the laboratory conditions and might not reflect everyday gait performance in the long term.

Footwear as a peripheral, proprioceptive factor and DBS as a central, neuromodulatory technique affect the human gait network at different sites. The spinal “central pattern generator” and the “MLR” are controlled by supraspinal networks and peripheral, sensory feedback from various somatosensory systems (Takakusaki, 2013). In PD, gait network activity is disturbed with activity changes at different sites (Grabli et al., 2012). It is interesting to what extent modulation at peripheral and basal ganglia sites within the gait network affects the clinical outcome.

Barefoot walking has been assessed extensively in the healthy younger and older population. One of the most consistent findings during unshod gait is a reduction of step length and an increase of cadence (Franklin et al., 2015). These findings could be observed in our patients with PD group independent of the DBS mode or gait task, and thus, footwear impacted generally step length and cadence. There are several hypotheses on this kinematic finding when walking with shoes. On the one hand, the increased distal mass of the foot when wearing footwear might induce a higher pendulum effect and inertia during the swing phase (Oeffinger et al., 1999). Another hypothesis is the modulation of sensory feedback by footwear (Franklin et al., 2015) since cutaneous receptors in the feet are assumed to play an important role in gait and postural control (Viseux et al., 2019) according to the gait network model with sensory afferents projecting and modulating the spinal central pattern generators.

To summarize considerations about footwear, it is difficult to advise patients with PD to walk barefoot or with shoes at home or during rehabilitative training sessions, since both gait modes have their specific advantages. Barefoot walking might enhance proprioceptive feedback besides its favorable foot mechanics, foot awareness, or strengthening. Appropriate footwear seems to stabilize gait and can be scientifically used as a vehicle for monitoring gait or to improve FOG by cueing (Barthel et al., 2018b). In terms of gait analysis, one needs to consider footwear as a factor in a longitudinal study with repeated measurements over time.

The effects of DBS have been assessed quite intensively. We found that DBS induced step length increment, gain of relative single support time, and reduction of gait asymmetry depending on the gait task. These quantitative measures are supposed to reflect indirect biomarkers for the clinical phenomenon of FOG in the interictal phase (O'Day et al., 2020) and indicate potential effects of DBS on FOG.

In previous studies, the effect of STN DBS on gait and FOG was variable (Potter-Nerger and Volkmann, 2013), with gait improvement in about one-third of patients with PD, remaining effective for 3–5 years (Schlenstedt et al., 2017). Recent efforts have been made to stimulate simultaneously the STN and SN (STN+SN DBS) (Weiss et al., 2011a,b, 2013; Scholten et al., 2017). Although the different, simultaneous mechanisms of action of DBS at cellular, populational, and network level are still debated, the overall effect might be a “functional inhibition” since clinically DBS effects are comparable to those of the previous stereotactic lesions. STN+SN DBS was introduced based on the anatomical considerations of dense basal ganglia interconnections to brain stem centers *via* SNr (Nandi et al., 2002), which might play a major role as a final common pathway (Georgiades et al., 2019) in the mediation of gait symptoms and FOG. The inhibitory high-frequency co-stimulation of the SN (Weiss et al., 2011a,b, 2013) was proposed to release the excessive basal ganglia inhibitory tone on the MLR, which in turn mediates the actual gait program to spinal locomotor centers coordinating bilateral lower limb movements (Lewis and Shine, 2016). Another approach was the use of low-frequency DBS within the pedunculopontine nucleus (PPN), which was assumed to reactivate the pathologically suppressed PPN activity within the MLR (Jenkinson et al., 2009; Thevathasan et al., 2018); however, the clinical results remained inconsistent (Thevathasan et al., 2012; Bourilhon et al., 2021), so that this procedure remains an experimental approach.

In our cohort of patients with PD, we assessed STN+SN DBS in postoperative patients in gait tasks with low and high cognitive load. Our results revealed a favorable effect of STN+SN DBS on gait compared to STN DBS as described previously (Weiss et al., 2013). We found improvement in spatial and temporal gait characteristics with STN+SN DBS, which were emphasized in gait tasks with the increased cognitive load as performing dual tasks. This particular improvement in cognitive gait aspects by STN+SN DBS might be due to the role of SNr in cognitive processes since SNr is proposed to be involved in cognitive, attentional control of purposeful movements and gaze to enhance the valuable outcome of the selected action (Sato and Hikosaka, 2002). The projections of the SNr connect not only the caudate nucleus and superior colliculus but also the thalamocortical and brain stem nuclei. These nigral circuits are proposed to be involved in cognitive, attentional control of purposeful movements to enhance the success of the selected action. To further evaluate the beneficial effects of STN+SN DBS in clinical routine, multicenter studies with larger collectives are needed.

In summary, footwear and DBS affect spatial and temporal kinematics of gait. The effect of footwear with the enhancement of step length and decrease of cadence needs to be considered when planning longitudinal studies or rehabilitative training settings. DBS improves gait kinematics, particularly STN+SN DBS is useful in the improvement of gait characteristics in conditions with increased cognitive load. Clinical benefits, side effects, and changes of quality of life in the long term still need to be assessed in more detail.

## Data Availability Statement

The raw data supporting the conclusions of this article will be made available by the authors, without undue reservation.

## Ethics Statement

The studies involving human participants were reviewed and approved by Ethikkommission der Ärztekammer Hamburg. The patients/participants provided their written informed consent to participate in this study.

## Author Contributions

For the research project, AG and MP-N contributed to the conception. MH, AG, UH, CG, WH, CM, and MP-N organized the project. MH, AG, UH, WH, CM, and MP-N contributed to the execution of the project. For statistical analysis, AG and MP-N contributed to the design; MH and AG contributed to the execution; MH, AG, and MP-N contributed to the review and critique. For the manuscript, MH and MP-N contributed to the writing of the first draft; MH, AG, UH, CG, WH, CM, and MP-N contributed to the review and critique. All authors contributed to the article and approved the submitted version.

## Funding

This study was financially supported by the DFG (SFB 936/C8: CM and MP-N; SFB 936/C1: CG).

## Conflict of Interest

AG received travel reimbursements from Medtronic Inc. CG reports personal fees and other from Bayer Healthcare and Boehringer Ingelheim and personal fees from Acticor Biotech, Sanofi Aventis Amgene, and Prediction Bioscience. CM served as medico-scientific consultant to Abbott. WH received lecture fees and honoraria for serving on advisory boards and travel grants from Boston Scientific, Medtronic, and Abbott. MP-N received lecture fees from Abbott and Licher, travel grants from AbbVie, and served as consultant for Medtronic, Boston Scientific, and AbbVie. The remaining authors declare that the research was conducted in the absence of any commercial or financial relationships that could be construed as a potential conflict of interest.

## Publisher's Note

All claims expressed in this article are solely those of the authors and do not necessarily represent those of their affiliated organizations, or those of the publisher, the editors and the reviewers. Any product that may be evaluated in this article, or claim that may be made by its manufacturer, is not guaranteed or endorsed by the publisher.

## Supplementary Material

Supplementary Figure 1 Repeated measures correlations. This method was applied to assess consistencies between the gait parameters velocity **(A–C)**, step length **(D–F)**, cadence **(G–I)**, and single support **(J–L)** in the dual task with shoes and the clinical scores FOG, Berg Balance, and MDS-UPDRS part III at the three DBS conditions (OFF DBS, STN, and STN+SN DBS). Repeated measures correlation (i.e., rmcorr) is a statistical technique for determining the common within-individual association for paired measures assessed on more occasions for multiple individuals (Bakdash and Marusich, 2017). The rmcorr accounts for non-independence among observations using the analysis of covariance (ANCOVA) to statistically adjust for interindividual variability. Unlike simple correlations, rmcorr does not violate the assumption of independence of observations. Colors coded are the single patients at the three DBS conditions. The separate parallel lines show the rmcorr fit for each individual patient. The sign of the rmcorr coefficient (i.e., positive or negative) is indicated by the direction of the common regression slope plotted as an interrupted line. Inset values give the statistics for the corresponding rmcorr.
