# Supplementary figures and images for: Comparison of Shod and Unshod Gait in Patients With Parkinson's Disease With Subthalamic and Nigral Stimulation
Source: Front Hum Neurosci. 2022 Jan 12;15:751242. doi: 10.3389/fnhum.2021.751242 (PMC8790533; doi:10.3389/fnhum.2021.751242)

# Dual task with shoes

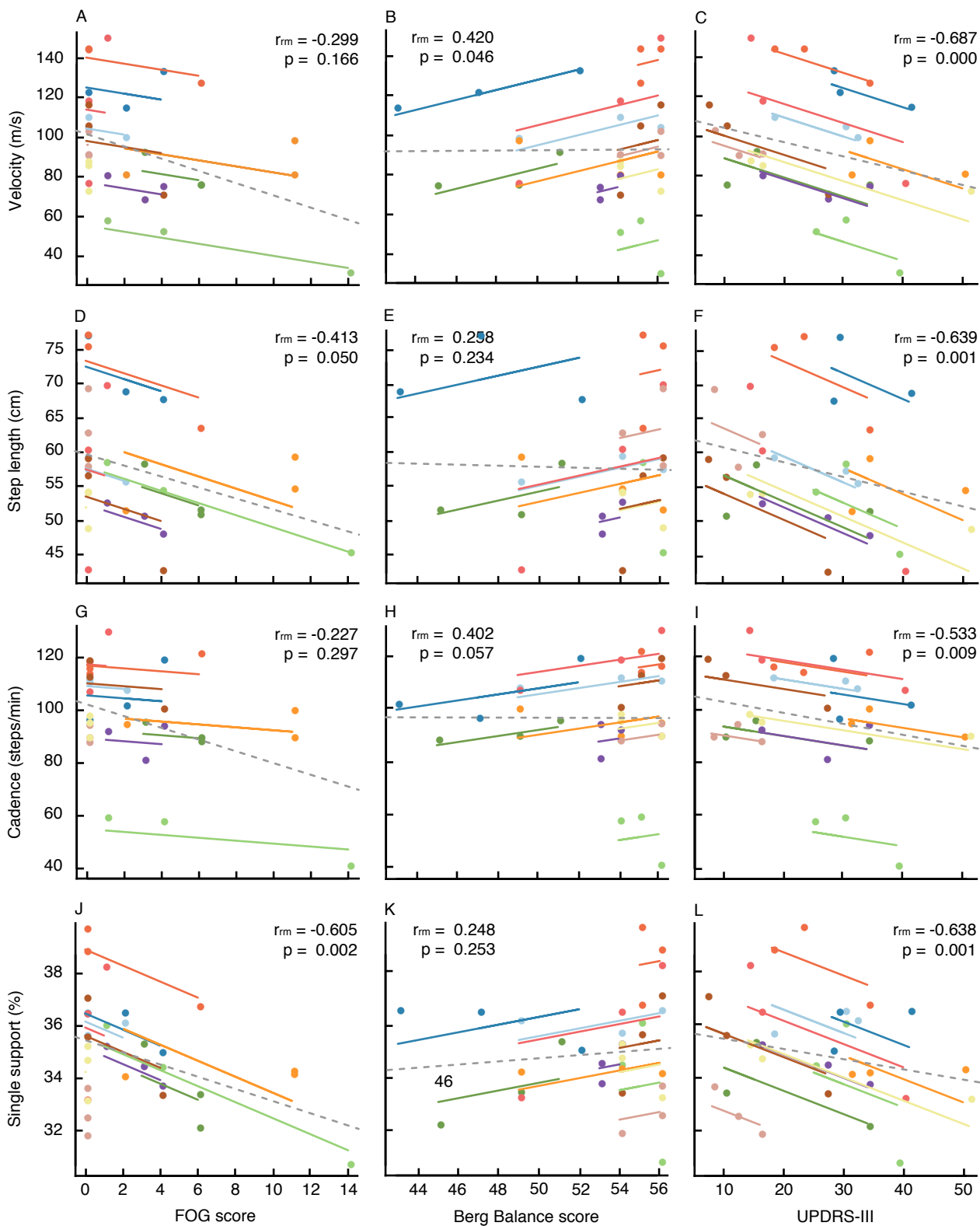

Supplement: Supplementary Figure 1 — Repeated measures correlations. This method was applied to assess consistencies between the gait parameters velocity (A–C), step length (D–F), cadence (G–I), and single support (J–L) in the dual task with shoes and the clinical scores FOG, Berg Balance, and MDS-UPDRS part III at the three DBS conditions (OFF DBS, STN, and STN+SN DBS). Repeated measures correlation (i.e., rmcorr) is a statistical technique for determining the common within-individual association for paired measures assessed on more occasions for multiple individuals (Bakdash and Marusich, 2017). The rmcorr accounts for non-independence among observations using the analysis of covariance (ANCOVA) to statistically adjust for interindividual variability. Unlike simple correlations, rmcorr does not violate the assumption of independence of observations. Colors coded are the single patients at the three DBS conditions. The separate parallel lines show the rmcorr fit for each individual patient. The sign of the rmcorr coefficient (i.e., positive or negative) is indicated by the direction of the common regression slope plotted as an interrupted line. Inset values give the statistics for the corresponding rmcorr. [file Data_Sheet_1.PDF]
